# Visual evoked potentials in the horse

**DOI:** 10.1186/s12917-016-0743-3

**Published:** 2016-06-21

**Authors:** L. Ström, B. Ekesten

**Affiliations:** Department of Clinical Sciences, Swedish University of Agricultural Sciences, PO Box 7054, SE-750 07 Uppsala, Sweden

**Keywords:** Visual evoked potentials, Visual cortical evoked potentials, Flash visual evoked potentials, VEP, FVEP, Horse, Equine, Visual impairment

## Abstract

**Background:**

Electrical potentials generated in the central nervous system in response to brief visual stimuli, flash visual evoked potentials (FVEPs), can be recorded non-invasively over the occipital cortex. FVEPs are used clinically in human medicine and also experimentally in a number of animal species, but the method has not yet been evaluated in the horse. The method would potentially allow the ophthalmologist and equine clinician to evaluate visual impairment caused by disorders affecting post-retinal visual pathways. The aim was to establish a method for recording of FVEPs in horses in a clinical setting and to evaluate the waveform morphology in the normal horse.

**Methods:**

Ten horses were sedated with a continuous detomidine infusion. Responses were recorded from electrodes placed on the scalp. Several positions were evaluated to determine suitable electrode placement. Flash electroretinograms (FERGs) were recorded simultaneously. To evaluate potential contamination of the FVEP from retinal potentials, a retrobulbar nerve block was performed in two horses and transection of the optic nerve was performed in one horse as a terminal procedure.

**Results:**

A series of positive (P) and negative (N) peaks in response to light stimuli was recorded in all horses. Reproducible wavelets with mean times-to-peaks of 26 (N1), 55 (P2), 141 (N2) and 216 ms (P4) were seen in all horses in all recordings. Reproducible results were obtained when the active electrode was placed in the midline rostral to the nuchal crest. Recording at lateral positions gave more variable results, possibly due to ear muscle artifacts. Averaging ≥100 responses reduced the impact of noise and artifacts. FVEPs were reproducible in the same horse during the same recording session and between sessions, but were more variable between horses. Retrobulbar nerve block caused a transient loss of the VEP whereas transection of the optic nerve caused an irreversible loss.

**Conclusions:**

We describe the waveform of the equine FVEP and our results show that it is possible to record FVEPs in sedated horses in a clinical setting. The potentials recorded were shown to be of post-retinal origin. Further studies are needed to provide normative data and assess potential clinical use.

## Background

Techniques currently available to evaluate visual impairment in the horse are limited to reflex testing, falling cotton-ball testing, ability to navigate an obstacle course and evaluation of findings detected during a routine ophthalmic examination. The results from these tests can be difficult to interpret and visual dysfunction may have to be severe before it can be diagnosed. Objective methods for evaluation of the function of visual pathways, such as visual evoked potentials (VEPs), would be a useful addition to the armamentarium of the veterinary ophthalmologist and equine clinician, to detect and evaluate disorders affecting post-retinal pathways in this species.

In 1954, Adrian & Matthews [1] reported that electrical potentials generated by neurons in the central nervous system in response to visual stimuli, VEPs, can be recorded from electrodes placed over the human occipital cortex. Dawson [2] developed a superimposition technique, called signal averaging, to extract the VEP waveform from the electroencephalogram (EEG) and subsequently, Cigánek [3] described the waveform morphology of the human VEP. In the years following further work was performed to explore the VEP in human beings, improve the technique and implement recording of VEPs as a clinical, diagnostic aid.

Diseases or trauma affecting neurons in the retina or the post-retinal visual pathways can alter the waveform of the VEP. Therefore this technique can be used to investigate the functional integrity of post-retinal visual pathways, including the optic nerves, optic tract and optic radiation to the visual cortex. The method is used as a complement to the electroretinogram (ERG) and allows functional assessment of the visual pathways, while imaging techniques, such as magnetic resonance imaging (MRI) and computer tomography (CT), mainly are used to obtain information about structural integrity in patients. VEPs are used in human medicine to evaluate visual impairment due to several pathological conditions, such as cortical trauma or anoxia, meningitis, optic neuritis, optic neuropathies, demyelinating diseases and compression of post-retinal pathways due to tumors [4].

The International Society for Clinical Electrophysiology of Vision (ISCEV) regularly updates the human standard protocols for clinical electrophysiological examinations [5]. Guidelines are also issued by the American Clinical Neurophysiology Society [6]. In human patients, VEPs are usually recorded non-invasively using skin electrodes on the scalp overlying the visual cortex. In routine testing the VEP scalp electrodes are normally placed according to either the International 10/20 system [7] or the Queen Square System [8]. Midline electrodes over the visual cortex are used to assess prechiasmal function, while additional lateral electrode positions are used to assess chiasmal and postchiasmal dysfunction.

Usually one eye at a time is stimulated by flashes of white light (flash-VEP; FVEP) or a reversing, isoluminant pattern (pattern-VEP; PVEP). In human subjects, PVEPs are preferred in most clinical situations due to less variability than the FVEPs and PVEPs will also enable a better quantitative assessment of visual function. However, when using a pattern stimulus it is critical that the subject focuses on the pattern and in infants and patients with poor cooperation, poor fixation or poor acuity, the FVEP can be more useful [5].

While VEPs have been evaluated in a number of animal species such as the dog [9, 10], cat [11–13], cow [14], pig [15], sheep [16] and a large number of laboratory animal species [17], the use of VEP in the horse has only been very scantily described [18]. A simple protocol for recording the equine VEP in a clinical setting would be a useful tool to assess function of post-retinal pathways in this species and also of use in the investigation of neurological disorders.

## Methods

The aim of this study was to establish a method for recording FVEP in horses in a clinical setting and to evaluate the waveform morphology in the normal horse. Electrode positions and the number of averaged responses needed to produce robust and reproducible VEP waveforms were also assessed. Additionally, recording of FVEPs after retrobulbar nerve block and transection of the optic nerve was performed to evaluate possible contamination of the FVEP by retinal potentials (ERGs).

### Horses and procedures

Ten Swedish Standardbred Trotters, seven mares and three geldings, aged 3 to 19 years (median age: 11 years) were included in the study (Table 1). All horses were deemed to be healthy on the basis of a physical examination prior to the recordings. The regional Ethical Committee (Uppsala Djurförsöksetiska nämnd, Sweden, dnr C254/10 and dnr C39/12) approved the use of the horses in the study and the experiments were carried out following the guidelines of the ARVO guidelines for animal experimentation.

**Table 1:** FVEPs and FERGs were performed in ten healthy Swedish Standardbred Trotters

Horse	Age at examination (years)	Gender	Eye(s)	ERG	Number of averages	Electrode positions	Lateral positions	Transection	Retro bulbar nerve block	Reproducibility
A	19	M	OD	X	4–196	P_z-0_ P_z-15_ P_z-30_ P_z- 45_ P_z-60_	P_1_ P_2_ P_3_ P_4_	-	-	X
B	10, 11	G	OS	X	144	P_z-0_ P_z-15_ P_z-30_ P_z- 45_ P_z-60_	P_1_ P_2_ P_3_ P_4_	-	-	X
C	16, 17	M	OD	X	144	P_z-0_ P_z-15_ P_z-30_ P_z- 45_ P_z-60_	P_1_ P_2_ P_3_ P_4_	-	-	-
D	16, 17	M	OS (OD)	X	144	P_z-0_ P_z-15_ P_z-30_ P_z- 45_ P_z-60_	P_1_ P_2_ P_3_ P_4_	-	-	X
E	10, 11	M	OD	X	4–196	P_z-0_ P_z-15_ P_z-30_ P_z- 45_ P_z-60_	P_1_ P_2_	-	-	X
F	5	G	OS	X	4–196	P_z-45_	P_1_ P_2_ P_3_ P_4_	-	-	X
G	17	M	OS	X	144	P_z-45_	P_1_ P_2_	-	-	-
H	3	M	OS (OD)	X	144	P_z- 45_	P_1_ P_2_	-	-	-
I	4	M	OS (OD)	X	144	P_z-45_	P_1_ P_2_	X	X	-
J	5	G	OD (OS)	X	144	P_z-45_	P_1_ P_2_	-	X	-

Recordings from one, randomly selected eye were performed in six horses and from both eyes in four horses. “X” indicates that a certain procedure was performed. There were no statistical differences comparing eyes in this study

In five horses (horses A-E) five electrode positions in the midline, and two to four lateral positions were evaluated (Table 1). In the remaining five horses (horses F-J), one position in the midline and two to four lateral positions were evaluated. FVEPs were recorded at two separate sessions in five horses (horses A-B and D-F). The minimum number of responses averaged to obtain a waveform with clearly distinguishable wavelets was evaluated in three horses (horses A, E and F). For comparison between eyes in the same horse, both eyes were evaluated in horses D and H-J. Unilateral retrobulbar nerve blocks were performed in two horses (horses I and J) and transection of the optic nerve was performed in one horse (horse I). This was performed as a terminal procedure.

### Ophthalmic examination

An ophthalmic examination including a cotton-ball test, reflex testing, slit-lamp biomicroscopy (Kowa SL-15; Kowa Co.Ltd., Tokyo, Japan), direct and indirect ophthalmoscopy (Heine Beta 200 and Heine Omega 500; Heine Optotechnik, Herrsching, Germany) and tonometry (TonoVet^®^, Icare Finland Oy, Helsinki, Finland) was performed in all horses. Furthermore, all horses were evaluated in a simple obstacle course, with five obstacles, under room-light conditions to assess vision prior to the recordings. No findings indicating visual impairment, ocular or neurological disease were observed in any of the horses.

### Preparations

Pupils were dilated with 1–2 drops of tropicamide (Mydriacyl, 0.5 %, Alcon, Stockholm, Sweden). Recordings were started when pupils were fully dilated. The size of the pupils was monitored repeatedly during the recording sessions. The cornea was kept moist throughout the experiment by repeated instillation of artificial tears (ZilkEye, Evolan Pharma AB, Stockholm, Sweden).

The horses were sedated with a bolus injection of detomidine, 0.01 mg/kg (Domosedan vet, 10 mg/ml, Orion Pharma Animal Health, Sollentuna, Sweden) intravenously, followed by maintenance of sedation throughout the recording sessions using a continuous intravenous infusion of 2 % detomidine in physiologic saline solution (Natriumklorid, 7 mg/ml, Fresenius Kabi, Uppsala, Sweden) (2.0–2.6 mg detomidine hydrocloride/100 kg*h). The depth of sedation was monitored continuously and the infusion rate was adjusted to maintain a stable level of sedation where the horses kept their heads resting heavily on a padded headstand without becoming unsteady.

Akinesia and partial analgesia of the eyelids was induced with subcutaneous injections of 2.5 ml mepivacaine (Carbocain, 20 mg/ml, AstraZeneca, Södertälje, Sweden) over the auriculopalpebral and supraorbital nerves. A local infiltration of mepivacaine (Carbocain, 20 mg/ml, AstraZeneca, Södertälje, Sweden) was performed at each electrode position to reduce muscle contractions that potentially could give rise to artifacts in the FVEP. The cornea was anaesthetized with topical application of 2–3 drops of tetracaine (Tetrakain Chauvin, 1 %, Bausch & Lomb Nordic AB, Stockholm, Sweden).

**Fig. 1:**
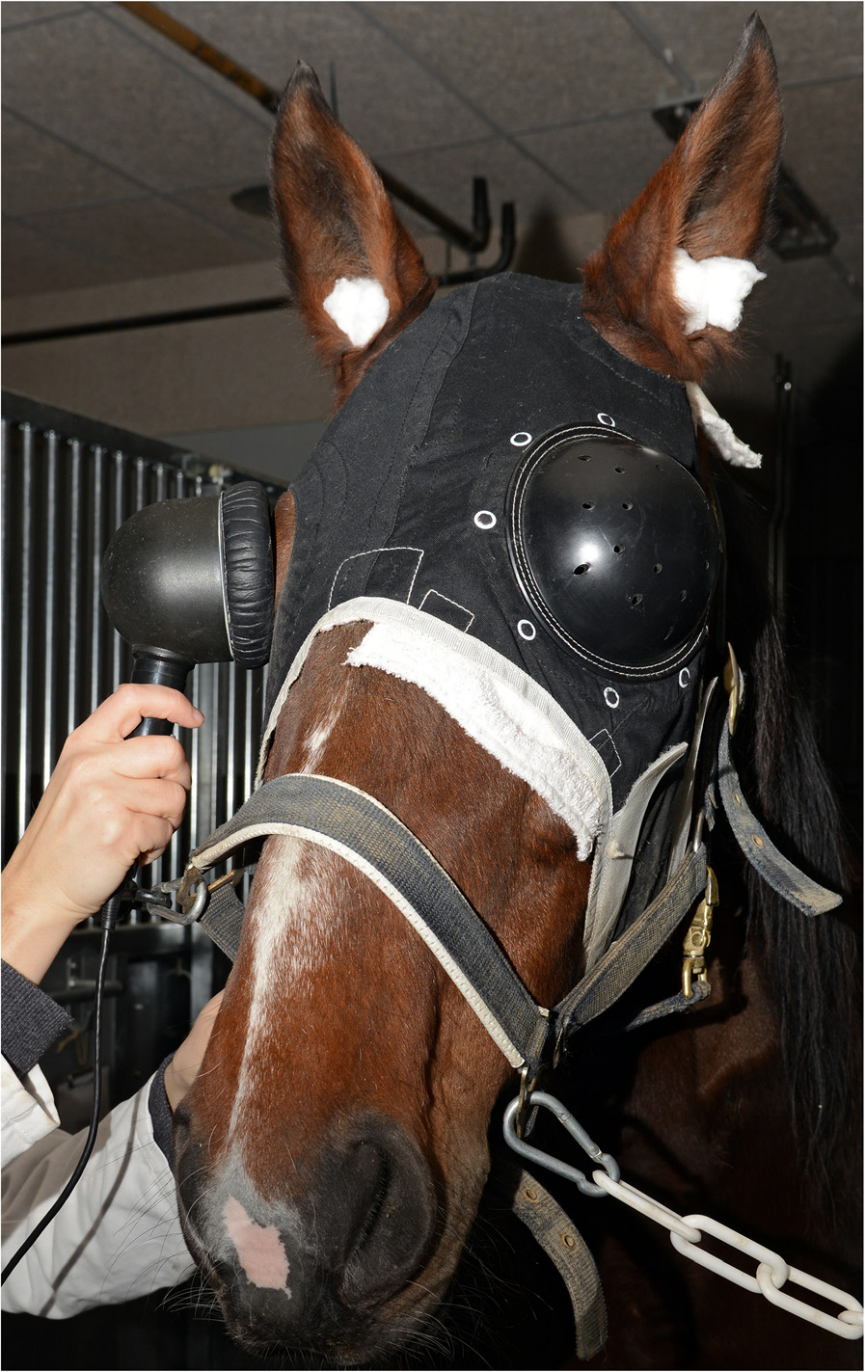
FVEP in a horse using a handheld flash photostimulator. An opaque plastic eye shield covers the fellow eye and cotton wads in the ears are used to reduce auditory stimuli that could influence the level of consciousness

The contralateral eye was covered by a black, opaque plastic eye shield during recordings (Fig. 1). Wads of cotton were stuffed in the ear canals to reduce noise-induced artifacts.

### Retrobulbar nerve block and transection of the optic nerve

Horses I and J were prepared as for a transpalpebral enucleation in the standing, sedated horse [19, 20]. A single dose of flunixin meglumine (Flunixin N-vet, 50 mg/ml, N-vet, Uppsala, Sweden), 1.1 mg/kg, was administered intravenously pre-operatively. Akinesia of the eyelids, anesthesia at electrode positions and topical anesthesia were performed and sedation maintained as previously described. FERGs and FVEPs were recorded. A retrobulbar nerve block was performed as has been described by Gilger & Davidson [21] and the surgical site was infiltrated with mepivacain (Carbocain, 20 mg/ml, AstraZeneca, Södertälje, Sweden). Transection of the optic nerve was performed (horse I) 45 min after the retrobulbar nerve block. FERGs and FVEPs were recorded and rebound tonometry performed before and after the retrobulbar block in both horses, as well as before and after transection of the optic nerve in horse I. Both horses (I and J) were euthanized after the recordings in accordance with the ethical approval.

### Electrophysiological testing

Light-adapted FERGs and FVEPs were recorded simultaneously. The recordings were performed in an examination room at the horse clinic of the University Animal Hospital, SLU, Uppsala. All electronic equipment not required for the study was disconnected to avoid mains current interference, but no other adjustments were made to the room.

A handheld dome-shaped photostimulator (Retiport mini-Ganzfeld, Acrivet GmbH, Hennigsdorf, Germany) with a background light intensity of 25 cd/m^2^ and a flash intensity of 3 cd/m^2^/s was used to light-adapt and stimulate the retina (Fig. 1). The stimuli were presented at a frequency of 1.09 Hz. The sweep duration was 500 ms post-stimulus. The room was kept dark during recordings to avoid interference by stray light.

Responses were amplified (amplifier set to x10^4^), band-pass filtered (0.1–300 Hz for FERGs and 1–100 Hz for FVEPs), stored and analyzed using an Acrivet RETI*port* (Acrivet GmbH, Hennigsdorf, Germany, software version 5.9.7.0) and a laptop computer.

### FERGs

Light-adapted FERGs were recorded using corneal electrodes (Gold-foil corneal electrodes, CH electronics, Bromley, UK). Metal corkscrew electrodes (Stainless Steel Disposable Corkscrew Electrode, Cephalon A/S, Nørresundby, Denmark) served as ground and reference electrodes and were placed at the forehead and approximately 3 cm caudal to the lateral canthus, respectively. Electrode impedance was kept below 3 kΩin all FERG recordings.

### FVEPs

**Fig. 2:**
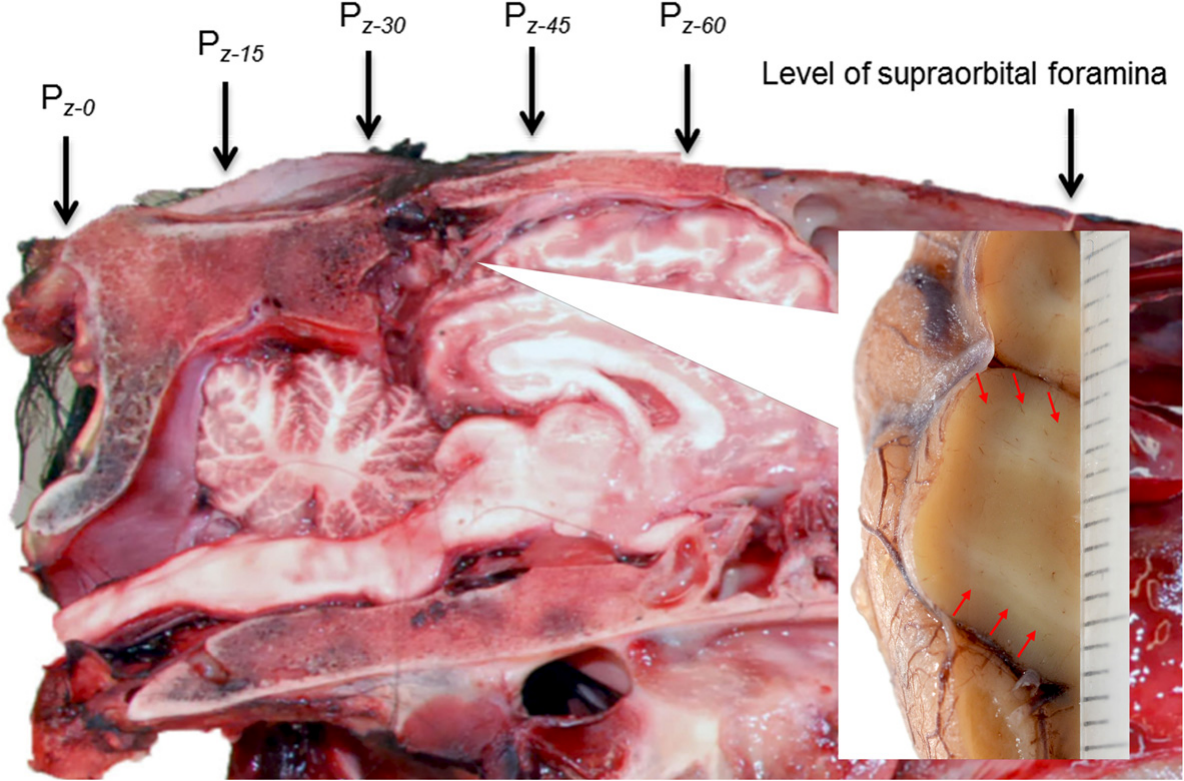
The evaluated electrode positions along the midline shown relative to the equine brain. The subscript. “z” denotes a position in the sagittal midline and the numerical subscript reflects a percentage of the distance between the nuchal crest and the connecting line between the supraorbital foramina. The insert shows the approximate position of the striate cortex, or primary visual cortex. Red arrows indicate the stria of Gennari, which represents myelinated axons from the lateral geniculate body terminating in the gray matter in the primary visual cortex

**Fig. 3:**
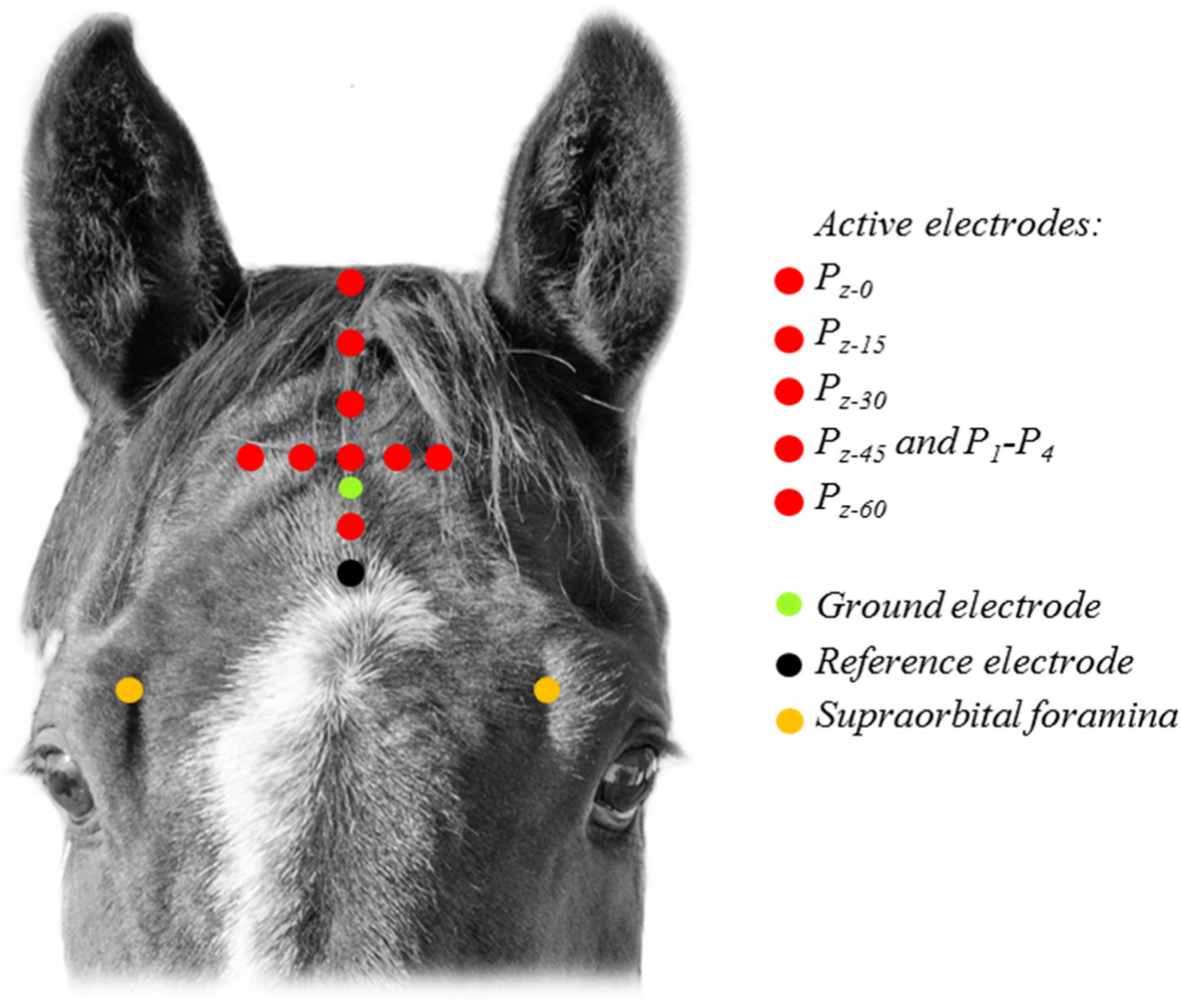
The first active electrode was placed on the nuchal crest in the midline (P_z-0_). The following positions along the midline were placed in front of the nuchal crest at regular intervals (P_z-0_ to P_z-60_). Lateral positions, P_1_ to P_4_, were evaluated with electrodes placed two and four centimeters lateral to the midline electrode at P_z-45_. Photo: Dr Måns Michanek

Electrode positions were essentially selected based on a combination of the International 10–20 system [7] and Queen Square System [8] for human beings. Figure 2 shows the position of evaluated electrode positions in the midline in relation to the equine brain and the primary visual cortex heralded by the stria of Gennari in our postmortem specimens. The electrodes were placed at distances relative to bony landmarks on the head to adjust for head size. The distance between the nuchal crest and a line connecting the supraorbital foramina was measured and the electrodes were positioned at preselected percentages along this distance (Fig. 3), where the nuchal crest was the starting point (0 %) and the level of the supraorbital foramina was at 100 %. The first active electrode was placed on the nuchal crest in the midline (P_z-0_; z = in the sagittal midline, 0 = 0 % rostral to the nuchal crest). The following positions of active electrodes along the midline were placed in front of the nuchal crest at intervals of 15 % (P_z-0_ to P_z-60_). Lateral positions, P_1_ to P_4_, were evaluated with electrodes placed two and four centimeters lateral to the midline electrode at P_z-45_. The ground and reference electrodes were positioned in the midline at 50 % and 70 %, respectively, of the distance from the nuchal crest and a line connecting the supraorbital foramina. Electrode impedance was kept below 2 kΩ in all FVEP recordings.

Averaged responses of 4, 16, 32, 64, 100, 144 and 196 sweeps were evaluated in horse A, E and F. The duration of a recording was therefore 3.7, 15, 29, 59, 92, 132 and 180 s respectively (frequency of 1.09 Hz). Averaged responses of 144 sweeps were evaluated in the remaining horses (horses B-D and G-J) and the duration of each averaged response was 2.2 min (132 s).

Monocular recordings were obtained from one randomly selected eye in all horses. In addition to this, recordings from the fellow eye were obtained in four horses (horses D and H-J to be able to enable comparison of recordings between eyes of the same horse.

### Evaluation of ERGs and VEPs

**Fig. 4:**
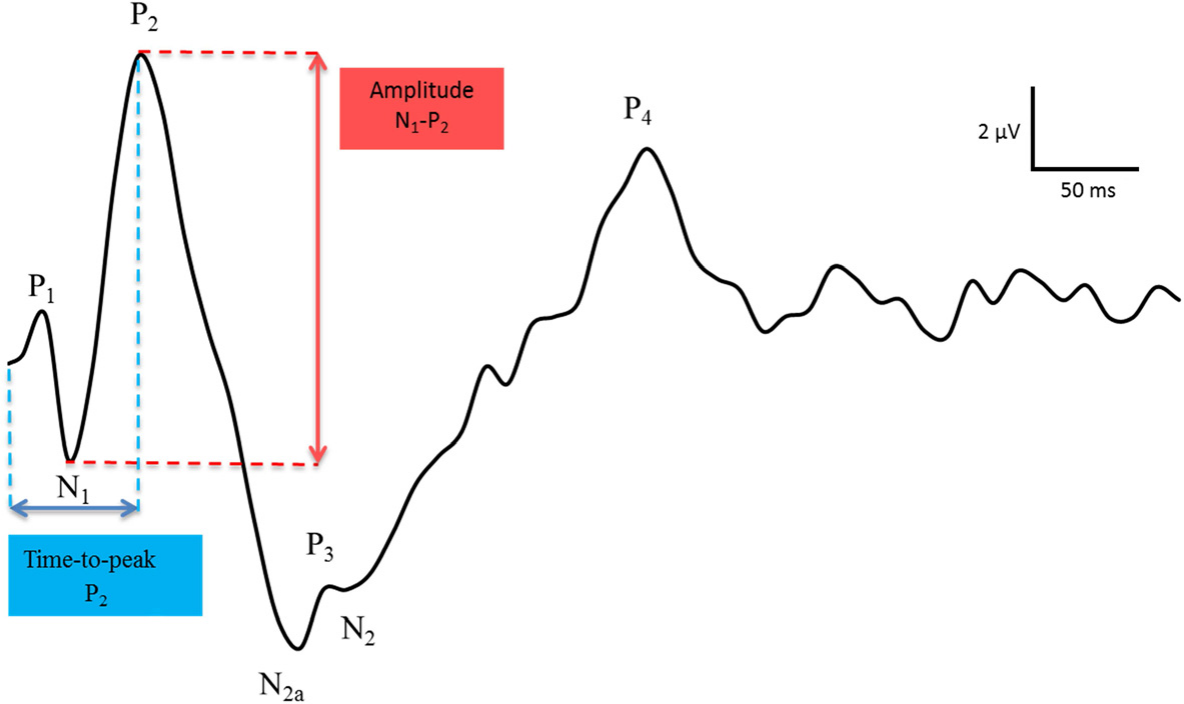
Measurements of amplitudes and times-to-peak in the equine FVEP. Amplitudes were measured from the peak or trough of wavelet preceding the trough or peak of interest. Time-to-peak measurements were made from the onset of the stimulus to the maximum amplitude of the peak or trough

FERG a- and b-wave amplitudes and times-to-peak (latencies) were measured according to convention [22]. The wavelets in the recorded FVEP waveform were named according to the nomenclature by Harding [23], a classification that was adopted in veterinary neurophysiology by Strain et al. in 1986 [14]. FVEP wavelet amplitudes and times-to-peak were measured as shown in Fig. 4. Results from recordings before and after retrobulbar nerve block (horse I-J) and transection of the optic nerve (horse I) were compared to evaluate if retinal potentials were recorded at the electrode positions on the scalp, thus contaminating the waveform, or if the recorded FVEP only represented post-retinal potentials.

### Data analyses

Descriptive data; means, standard deviations (SDs) and ranges are reported for FVEP wavelet peak-to-peak amplitudes (μV) and times-to-peaks (ms). Data were analyzed using the non-parametric Kruskal-Wallis one-way analysis of variance by ranks and by regression analysis. Levene´s test was used to assess the equality of variances between groups. P-values ≤0.05 were considered statistically significant. Bonferroni correction was used to adjust the p-values when multiple comparisons were performed. Statistical analysis was performed with JMP^®^ Pro 11 (Version 11.0.0, SAS Institute Inc., Cary, NC).

## Results

**Fig. 5:**
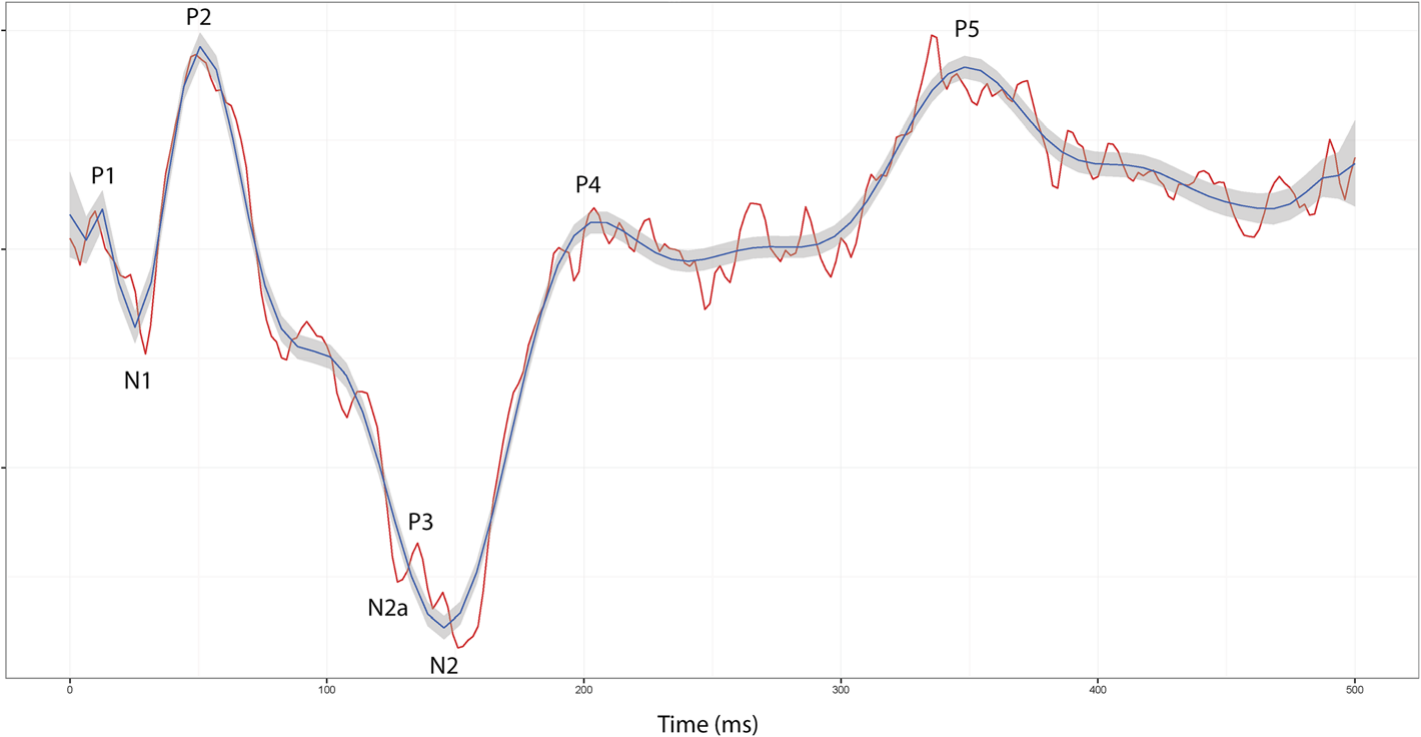
Equine FVEP waveform. An equine FVEP waveform with typical peaks and troughs indicated (from horse B; position P_z-30_). The red curve shows one recording (144 averaged responses). Data from 40 recordings (with 144 responses each) are averaged into the blue curve and shown with a 95 % confidence interval. Vertical division = 5 μV

Reproducible FVEPs and light-adapted FERGs were readily recorded from all ten, sedated horses. It was not possible to obtain reproducible recordings with acceptable quality from non-sedated horses due to movement and muscle artifacts. The waveform of the equine FVEP consisted of three major positive, P2-P4, and two major negative wavelets, N1-N2 (Fig. 5). The first negative wavelet, N1 and the most prominent positive wavelet, P2, was present in all recordings in all horses, as well as the large negative N2-complex and subsequent positive P4-wavelet. An early positive peak, P1, with a time-to-peak between 12–20 ms, was present in all horses, but only in approximately 50 % of the recordings in each horse. A small positive wavelet, P3, splitting the N2-complex into two parts, N2a and N2, was observed in all horses and in 82 % of recordings on average. A late positive wavelet, P5, with a time-to-peak between 275–375 ms was present in 74 % of recordings in nine out of the ten horses. Times-to-peak and peak-to-peak amplitudes are shown in Table 2.

**Table 2:** FVEP amplitudes and times-to-peaks in ten horses with the active electrode at P_z-45_

		Time-to-peak (ms)								Peak-to-peak amplitude (μV)			
Horse	Eye	P1	N1	P2	N2a	P3	N2	P4	P5	N1-P2	P2-N2	N2a-P3	N2-P4
A	OD	13 ± 1.4	28 ± 1.2	59 ± 2.0	124 ± 3.5	137 ± 8.7	150 ± 4.6	273 ± 8.6	Missing	9.6 ± 1.0	11.9 ± 1.8	2.5 ± 1.2	8.4 ± 2.9
B	OS	12 ± 3.5	26 ± 1.1	54 ± 2.3	113 ± 4.2	123 ± 3.5	145 ± 8.8	218 ± 13.8	373 ± 31.4	7.4 ± 0.7	15.7 ± 3.6	0.4 ± 0.1	13.5 ± 4.9
C	OD	14 ± 0.1	28 ± 1.0	62 ± 2.5	104 ± 4.1	125 ± 5.6	153 ± 7.5	207 ± 13.8	354 ± 15.8	6.1 ± 1.2	5.9 ± 1.4	1.5 ± 1.0	3.7 ± 1.1
D	OS	18 ± 1.0	25 ± 2.0	47 ± 1.5	87 ± 12.0	107 ± 10.0	138 ± 8.8	200 ± 15.3	260 ± 7.2	2.2 ± 0.8	4.5 ± 1.0	1.5 ± 1.0	4.5 ± 1.1
E	OD	16 ± 3.5	29 ± 0.1	55 ± 4.0	99 ± 1.4	116 ± 2.8	153 ± 6.8	234 ± 24.6	375 ± 10.2	3.8 ± 1.5	4.6 ± 1.4	2.9 ± 1.1	4.5 ± 1.5
F	OS	12 ± 2.3	27 ± 1.4	54 ± 4.6	105 ± 4.6	122 ± 7.6	148 ± 6.4	213 ± 18.2	361 ± 26.2	7.3 ± 1.8	13.8 ± 3.9	0.4 ± 0.3	11.1 ± 3.7
G	OS	17 ± 1.2	30 ± 2.7	58 ± 3.0	104 ± 22.2	111 ± 18.9	137 ± 5.5	213 ± 13.3	355 ± 30.3	2.6 ± 0.6	6.2 ± 1.2	2.8 ± 4.7	8.3 ± 2.5
H	OD	20 ± 1.1	32 ± 5.9	57 ± 4.0	84 ± 28.9	95 ± 27.0	133 ± 7.9	202 ± 13.7	337 ± 32.7	2.5 ± 0.5	5.7 ± 1.5	0.9 ± 0.4	8.0 ± 2.6
I	OS	12 ± 3.1	17 ± 3.5	48 ± 2.2	103 ± 6.2	110 ± 8.2	130 ± 3.0	193 ± 7.1	305 ± 12.1	1.8 ± 0.2	3.5 ± 0.7	0.2 ± 0.1	4.5 ± 0.7
J	OD	6 ± 2.6	22 ± 5.9	52 ± 4.8	94 ± 12.5	107 ± 12.9	140 ± 6.8	206 ± 20.0	347 ± 19.8	2.2 ± 0.6	5.0 ± 1.5	4.8 ± 1.5	4.8 ± 1.7
95 % CI		12–14	26–28	55–57	99–105	113–120	137–142	213–224	324–345	4.3–5.5	6.1–7.8	1.3–2.3	5.9–7.4

A minimum of 5 recordings (144 averaged responses each) are included from each horse

Data are shown as means ± SDs

**Fig. 6:**
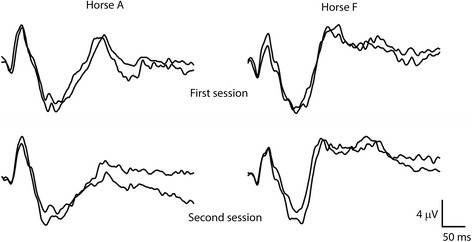
Reproducibility of equine FVEPs. FVEPs recorded from two horses (horses A and F) with the active electrode at P_z-45_. Two recordings from the first (top row) and second sessions several weeks later (bottom row) are shown

The waveform was reproducibly recorded and no significant differences in amplitudes or times-to-peaks were found when comparing recordings from the same recording session and at the following session (p > 0.05; Fig. 6). There were no significant differences in times-to-peaks or amplitudes regardless of eye stimulated.

**Fig. 7:**
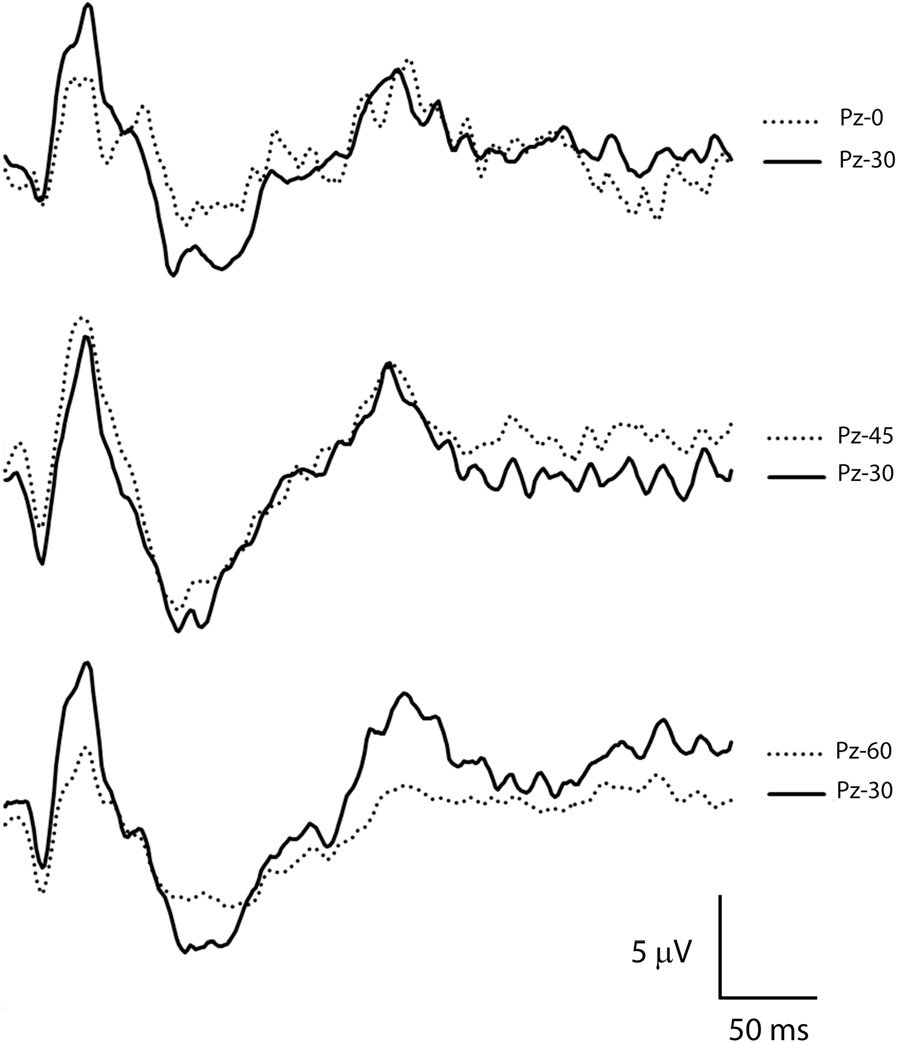
FVEPs from different electrode positions. The overall waveform morphology did not differ between electrode positions in the midline but amplitudes were significantly reduced at the most caudal (P_z-0_) and rostral (P_z-60_) positions and it was often more difficult to obtain a reasonably smooth waveform due to artifacts. Recordings from horse A

**Fig. 8:**
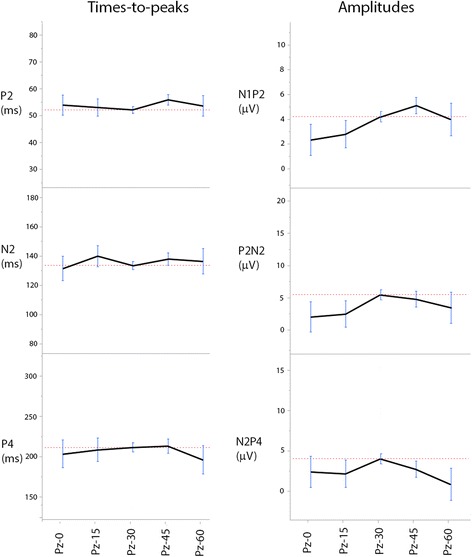
Amplitudes and times-to-peaks at different electrode positions. Amplitudes increased when moving the electrode position rostrally from the most caudal position at the nuchal crest, reaching maximum amplitudes at the positions P_z-30_ and P_z-45_. Moving further rostrally decreased the amplitudes. Times-to-peaks did not differ significantly between any of the positions tested in this material

The overall waveform morphology did not differ between the electrode positions in the midline (P_z-0_, P_z-15_, P_z-30_, P_z-45_ and P_z-60_) but the FVEP waveform was somewhat difficult to evaluate at the most caudal positions (Fig. 7). Average times-to-peak did not differ significantly between electrode positions but there were significant differences comparing amplitudes N1P2, P2N2 and N2P4 from recordings at P_z-0_ and P_z-60_ to recordings at P_z-30_ and P_z-45_ (p < 0.001). Amplitudes increased when moving the electrode position rostrally from the most caudal position at the nuchal crest, reaching maximum amplitudes at the positions P_z-30_ and P_z-45_ (Fig. 8). Moving further rostrally decreased the amplitudes.

At lateral positions (P_1_ to P_4_), both amplitudes and times-to-peak showed a greater variability compared to recordings in the midline but there were no significant differences between means of times-to-peaks and amplitudes in our material.

When averaging 4 and 16 responses, the waveform was very difficult to evaluate and most recordings were discarded. Times-to-peaks and amplitudes from recordings with 32 and 64 averaged responses did not differ statistically compared to recordings with 100, 144 and 196 averages, but the waveform was more challenging for the examiner to evaluate due to artifacts. By averaging no less than 100 responses, the background noise was reduced, which resulted in more easily discernible peaks and reduced number of peaks split by large artifacts.

**Fig. 9:**
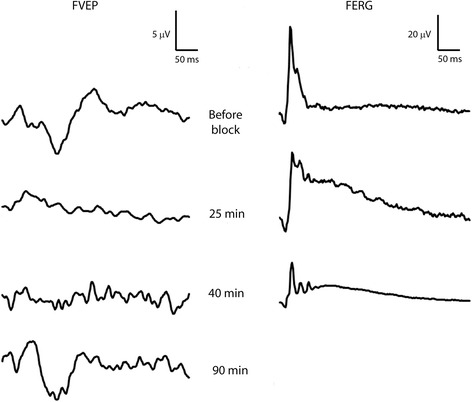
FVEPs after retrobulbar nerve block. FVEPs recorded at P_z-45_ in horse J from the right eye (left column) before retrobulbar nerve block (uppermost tracing), 25, 40 and 90 min after retrobulbar nerve block (lower tracings). Simultaneous FERG recordings from the right eye are shown in the right column

**Fig. 10:**
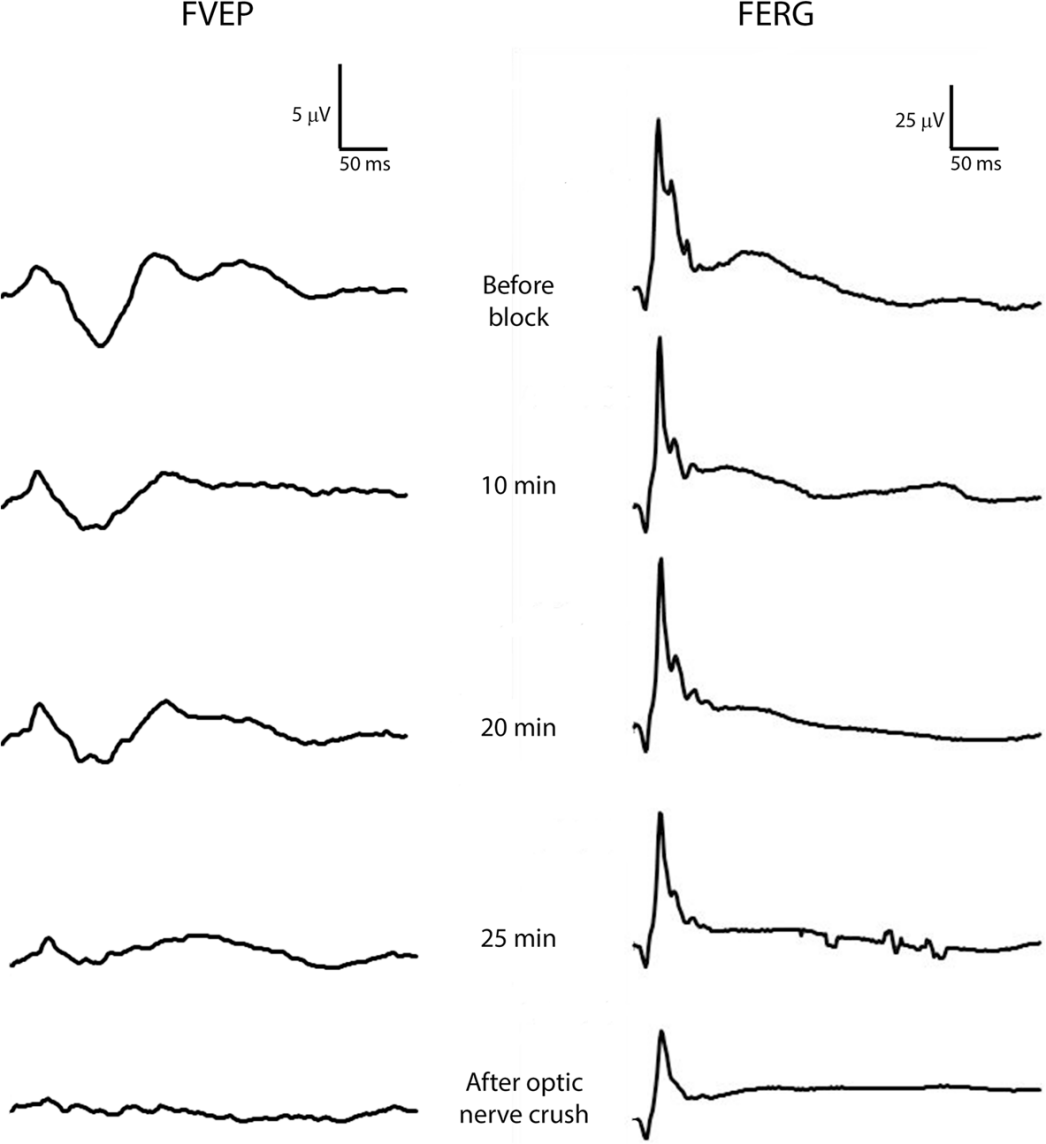
FVEPs after retrobulbar nerve block and transection of the optic nerve. FVEPs recorded at P_z-45_ in horse I from the right eye (left column) before retrobulbar nerve block (uppermost tracing), 10, 20 and 25 min after retrobulbar nerve block (middle rows) and after transection of the optic nerve (lowermost tracing). Simultaneous FERG recordings from the right eye are shown in the right column

The FVEP was affected by the retrobulbar nerve block (Figs. 9 and 10). FVEP amplitudes were gradually reduced in both horses (I and J) and after 25 min, only a minuscule remnant of P2 was discernible, despite that FERG a- and b-wave times-to-peaks were within normal limits and b-wave amplitudes were only marginally reduced. Intraocular pressures (IOPs) in the examined eye were 42 mmHg (horse J) and 56 mmHg (horse I) 25 min after the block (IOPs before retrobulbar block; 19 and 22 mmHg respectively). In horse J, the peaks and troughs of the equine FVEP were clearly visible again approximately 90 min after the nerve block, although FVEP amplitudes and times-to-peaks were not yet within normal limits. In horse I, the FVEP disappeared when the optic nerve was transected (Fig. 10). FERG a- and b-wave times-to-peaks were within normal limits after the transection, but amplitudes were somewhat reduced. The intraocular pressure was measured to 57 mmHg at this point. When the IOP rose above 70 mmHg due to retrobulbar hemorrhage and oedema (65 min after the nerve block), FERG a- and b-waves were also abolished.

Light-adapted FERGs were recorded from all eyes included in the study (Table 3). Times-to-peaks of the a- and b-waves were highly consistent both within and between horses while peak-to-peak amplitudes were more variable. Means for times-to-peaks of the a- and b-waves were 13 ± 1 ms and 30 ± 1 ms respectively. The mean a- and b-wave amplitudes were 10 ± 3 and 63 ± 15 μV respectively.

**Table 3:** Flash electroretinograms

	a-wave	b-wave
Implicit time (ms)	13 ± 1	30 ± 1
Amplitude (μV)	10 ± 3	63 ± 15

Light-adapted FERG amplitudes and times-to-peak in ten healthy horses. Data are shown as means ± SDs

## Discussion

VEPs were easily recorded in sedated horses under standard clinical settings and the overall waveform resembled those recorded in other mammalian species [5, 9–11, 13, 14, 16]. It has been shown in dogs that the signal recorded from VEP scalp electrodes can include a far-field conducted ERG [24, 25]. On the other hand, Sims et al. [9] have shown that transection of the optic nerve completely abolishes the light-adapted canine FVEP and that the recorded potentials are of post-retinal origin under light-adapted conditions. The horse has a large retina and the potentials generated are sizable. Thus it was important to evaluate if the equine FVEP recorded over the scalp consisted of post-retinal potentials only, or if a far-field conducted FERG was included. It is known in humans that a retrobulbar nerve block can cause temporary blindness due to a complete block of transmission in the optic nerve [26, 27] and we used retrobulbar anaesthesia and optic nerve transection to show that our recordings of the equine FVEP must be of post-retinal origin. Although the intraocular pressures were increased and FERG amplitudes were somewhat affected during the experiments, it has been shown in other species that increased intraocular pressures will result in reduced b-wave amplitudes [28–30], but simultaneous FVEP recordings will be more resistant to the pressure change [31]. Thus, it is highly unlikely that the increased intraocular pressure was causing the loss of the FVEPs in our recordings.

By using the FVEP we have also verified that the retrobulbar nerve block causes a transient loss of vision in the horse which should be considered when this method of anesthesia is employed for performing standing ocular surgery in this species.

Waveform morphology, times-to-peaks and peak-to-peak amplitudes were reproducible between recording sessions in the same horse and also similar between horses, although some differences in times-to-peaks and especially amplitudes were observed. It is well known from human beings [5] and other species [11] that the FVEP is highly variable between individuals. Four wavelets (N1, P2, N2 and P4) were observed in all FVEPs from all horses whereas additional wavelets were present in some recordings in all horses (P1, N2a and P3). A late, positive component (P5) was present in the majority of recordings in most horses. The differences in waveform morphology might represent the variability of the FVEP between individuals. However, in our study, these differences might also be due to varying depth of sedation, or that the individual horse might react differently to external disturbances in the clinical setting. The most reliable wavelets in the equine FVEP were the N1, P2, N2 and P4, which indicate that these peaks could be of use when the FVEP is employed as a diagnostic tool to diagnose and localize abnormal function in post-retinal visual pathways.

Choosing the suitable electrode position enhanced detection of the individual wavelets composing the equine FVEP. The wavelets became more obvious and distinct when the active electrodes were positioned well in front of the nuchal crest at electrode positions P_z-30_ and P_z-45_. These positions correspond well to the position of the occipital cortex and presumed anatomical location of the visual cortex in the horse [32], which we confirmed using the stria of Gennari as our landmark in postmortem specimens (Fig. 2). As expected, the electrode positions providing the largest amplitudes in the horse differ considerably from the positions recommended in the guidelines for clinical VEPs in human beings [5], reflecting the substantial anatomical differences between man and horse. An asymmetry was expected when recording the FVEP at ipsi- and contralateral positions due to the high degree of decussation of optic fibers in the horse of more than 80 % [33]. It was thus somewhat surprising that results from lateral positioning of the active electrodes were inconclusive, with regards to the separation of signals from the right and left hemispheres. The reason for this may be due to the close proximity to the ears of the horse. The lateral electrode positions were close to the base of the pinnae and the large muscles controlling ear movements in this species [34]. However, while muscle artifacts due to ear movements may have masked more subtle differences between lateral and midline positions, a method for obtaining FVEPs from the more lateral electrode positions without picking up artifacts from the ear muscles would be useful, because these positions may be important for differing between pre- and postchiasmal lesions in certain horses with visual impairment.

Sufficient and stable depth of sedation enhanced recording of FVEPs and FERGs in our study, which is in accordance with previous studies when FERGs were recorded in the standing, sedated horse [35–37]. Movement artifacts did occasionally overshadow a response, but the stable level of sedation obtained by continuous infusion of detomidine kept the horses sufficiently immobile to allow recording of reproducible FERGs and FVEPs. It was obvious that the depth of sedation influenced the amount of artifacts and the quality of recordings. When only light sedation was used, ear movements and head movements in response to sudden sounds caused muscle movement artifacts, which could wipe out the normal waveform. The use of earplugs reduced the episodes of increased alertness due to undesired auditory stimuli affecting the level of sedation. In summary, we conclude that a sufficiently stable level and depth of sedation can be accomplished by a bolus dose followed by a continuous infusion of detomidine. However, it is important to note that in human beings and other species, some sedatives have been shown to affect the VEP [38], causing reduced amplitudes and somewhat prolonged times-to-peak. To our knowledge, the specific effects of detomidine on the VEP has not yet been evaluated. It is reasonable to believe that amplitudes and times-to-peaks may be affected by sedation with alfa-2-agonists, but we were unable to obtain recordings with acceptable quality from non-sedated horses. Therefore, we recommend performing recordings under sedation. To be able to compare results between horses, it is important to continuously monitor the level of sedation during the recordings.

According to our results, at least 100 sweeps are needed in sedated horses to suppress electrical noise and obtain curves with easily discernible, reproducible peaks and troughs. This might be especially important for the inexperienced examiner. Overall, the equine FVEP appears to require more averaged responses than suggested by the ISCEV guidelines, which is likely to reflect that both the amount of potentially disturbing auditory stimuli in a busy equine clinic is higher than in a human ophthalmology or neurology clinic, and that our equine patients may be less cooperative and docile than the average adult human patient. However, it is important to note that 32–64 averaged responses in the horse may also produce acceptable results. Although there might be more noise and artifacts affecting the quality of the recordings, our results indicate that times-to-peaks and amplitudes will not be significantly affected when measured by an experienced examiner. The advantage of averaging fewer responses is that the time to perform the examination will be reduced, which might be important when recording the VEP in a critically ill patient.

The corkscrew electrodes used were applied without difficulties and were well tolerated by the horses and maintained good contact between electrodes and horse (impedance <2 kΩ) throughout the recordings. Other technical considerations include keeping the connecting wires as short as possible to avoid mains interference from electric equipment in the examination room and ensuring equipment not necessary for the procedure are unplugged to reduce sources of electromagnetic noise.

Although this work confirmed that the potentials in the equine FVEP are of post-retinal origin, the exact sources of the potentials producing each of the wavelets in the equine VEP are still unknown. The FVEP has been a diagnostic aid in human medicine for decades, but the origins of some wavelets seen in the human FVEP are still not clearly identified. Further studies are needed in the horse to evaluate the components of the equine VEP with the aim of establishing the anatomical location of lesions causing certain aberrations in the VEP waveform.

The first study of the human VEP in different age groups was performed by Dustman and Beck in 1969 [39] who showed that with increasing age, the human VEP alters in regards to times-to-peak and amplitudes, although the overall morphology stays the same throughout the adult life-span. Studies on the age-related development of VEPs have been performed in several animal species such as for example in cats [13], dogs [40], ruminants [16, 41, 42] and pigs [15]. As we have only few horses at each age in this study, further studies are needed to evaluate the normal variation of the equine VEP related to increasing age.

The FVEP in combination with concurrent FERGs provide an opportunity for objective, functional assessment of the post-retinal visual pathways in the standing, sedated equine patient. Equine FVEPs would be valuable for evaluating similar types of pathological conditions affecting the visual system as in human patients, such as cortical trauma, optic neuritis, tumors and demyelinating diseases. Additionally, FVEPs offer an opportunity to detect persistent visual impairment or blindness after retrobulbar nerve block, which has been reported as a rare complication in human patients [43]. As well the technique could be an important additional diagnostic tool to rule out visual impairment in cases of abnormal behavior.

## Conclusions

We have recorded FVEPs in healthy, sedated horses in a clinical setting. Active electrodes positioned at P_z-30_ to P_z-45_ provided a good signal-to-noise ratio and reproducible waveforms. Results from recordings after retrobulbar anaesthesia and transection of the optic nerve showed that the recorded responses were of post-retinal origin. Our technique for FVEP in the horse is relatively easily mastered and opens up for objective, functional assessment of the post-retinal visual pathways in this species.
